# Exercise training improves cardiac function, quality of life and exercise capacity in patients with dilated cardiomyopathy

**DOI:** 10.1186/1532-429X-13-S1-P349

**Published:** 2011-02-02

**Authors:** Cameron J Holloway, Joseph Suttie, Sairia Dass, Pete Cox, Hamish Jackson, Andrew W Johnson, Jane M Francis, Theodoros Karamitsos, Stefan Neubauer, Kieran Clarke

**Affiliations:** 1 University of Oxford, Oxford, UK

## Objective

To determine the effects of short term exercise training on cardiac metabolism and function, during rest and exercise, in patients with dilated cardiomyopathy (DCM).

## Background

Exercise training may play a beneficial role in patients with DCM, however the effects on cardiac function at rest and during exercise have not been defined.

## Methods

Patients with DCM (n = 15, age 58 ± 2 years), stable on medical therapy, were studied before and after 8 weeks of training for 20 minutes, 5 times per week on a home exercise bike. Cardiac volumes and function were measured using MR at 3T during rest and leg exercise. High energy phosphate metabolism was measured as the ratio of phosphocreatine to ATP (PCr/ATP) by ^31^Phosphorus magnetic resonance spectroscopy (MRS) at 3T. Quality of life scores were calculated using the Minnesota Heart Failure Questionnaire and 6 minute walk tests were used to determine exercise capacity. All assessments were repeated after the 8 week training period.

## Results

**Figure 1:**
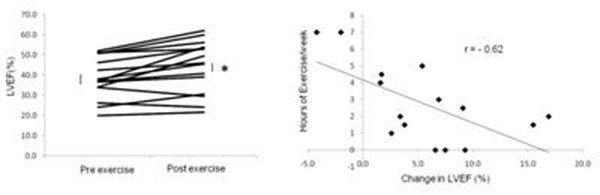


At baseline assessment, the average left ventricular ejection fraction (LVEF) was 38 ± 3%, which did not increase with leg exercise. After the 8 weeks of home exercise there was a 6% improvement in resting LVEF to 44 ± 3% (Figure, p <0.01) and an 8% reduction in end systolic volumes (p <0.05). Exercise training led to a further 8% improvement in cardiac LVEF (p < 0.05) during leg exericse in the MR scanner. There was a moderate negative correlation between subjects baseline exercise per week and change in LVEF during the trial (Figure 1, r = -0.62, p <0.05). Patients also had an 8% improvement in 6 minute walk test and a 27% improvement in Minnesota heart failure questionnaire scores after the exercise training (both p <0.01). Patient with DCM had impaired resting PCr/ATP, with no change after exercise training.

## Conclusions

Prior to exercise training, patients with DCM had no change in LVEF to leg exercise. A home exercise programme did not alter resting cardiac PCr/ATP, but improved cardiac function during rest and leg exericse, and increased exercise tolerance and quality of

life scores. The most sedentary patients prior to the exercise regime had the best improvement in cardiac function, suggesting cardiac muscle atrophy and deconditioning may play a role in the cardiac dysfunction seen in DCM. Exercise training may be particularly beneficial in this subgroup.

